# Supplementary material for: An open-source framework for large-scale, flexible evaluation of biomedical text mining systems
Source: J Biomed Discov Collab. 2008 Jan 29;3:1. doi: 10.1186/1747-5333-3-1 (PMC2276192; doi:10.1186/1747-5333-3-1)
Supplement: Additional file 1 — Evaluation platform source code. The additional file contains the software evaluation framework discussed in this paper. The most current release can be downloaded from SourceForge.net [1]. See the accompanying README file for instructions on installation and use. Also included in the distribution is a collection of UIMA wrappers for some commonly used BioNLP tools and annotated corpora. [file 1747-5333-3-1-S1.zip › bionlp_uima_repository-1.0.1/docs/api/src-html/edu/uchsc/ccp/uima/ae/nlp/ei/gene/KeX_AE.html]

```
001    /*
002     * Created on Sep 8, 2005
003     *
004     * TODO To change the template for this generated file go to
005     * Window - Preferences - Java - Code Style - Code Templates
006     */
007    package edu.uchsc.ccp.uima.ae.nlp.ei.gene;
008    
009    import java.util.Iterator;
010    import java.util.List;
011    
012    import org.apache.uima.analysis_engine.ResultSpecification;
013    import org.apache.uima.analysis_engine.annotator.AnnotatorConfigurationException;
014    import org.apache.uima.analysis_engine.annotator.AnnotatorContext;
015    import org.apache.uima.analysis_engine.annotator.AnnotatorContextException;
016    import org.apache.uima.analysis_engine.annotator.AnnotatorInitializationException;
017    import org.apache.uima.analysis_engine.annotator.AnnotatorProcessException;
018    import org.apache.uima.analysis_engine.annotator.JTextAnnotator_ImplBase;
019    import org.apache.uima.jcas.JCas;
020    
021    import edu.uchsc.ccp.uima.annotation.CCPDocumentInformation;
022    import edu.uchsc.ccp.uima.util.UIMA_Util;
023    import edu.uchsc.ccp.util.external.kex.KeXEISystem;
024    import edu.uchsc.ccp.util.nlp.annotation.AnnotationSet;
025    import edu.uchsc.ccp.util.nlp.annotation.TextAnnotation;
026    import edu.uchsc.ccp.util.nlp.mention.ClassMention;
027    
028    /**
029     * @author williamb Takes longer time to run the first time.. not sure why
030     */
031    public class KeX_AE extends JTextAnnotator_ImplBase {
032        private final boolean DEBUG = false;
033    
034        private KeXEISystem eiSystem;
035    
036        private String kexBaseDirectory;
037    
038        /**
039         * Name of configuration parameter that must be set to the path of the directory TnT will use for storing files generated in the gene
040         * annotation process.
041         */
042        public static final String PARAM_ABGENE_BASE_DIRECTORY = "KeXBaseDirectory";
043    
044        public void initialize(AnnotatorContext ac) throws AnnotatorInitializationException, AnnotatorConfigurationException {
045    
046            try {
047                kexBaseDirectory = (String) ac.getConfigParameterValue(PARAM_ABGENE_BASE_DIRECTORY);
048            } catch (AnnotatorContextException ace) {
049                ace.printStackTrace();
050                kexBaseDirectory = null;
051            }
052            System.err.println("Initializing KeX EI System ...");
053            eiSystem = new KeXEISystem(kexBaseDirectory);
054            super.initialize(ac);
055        }
056    
057        /*
058         * (non-Javadoc)
059         * 
060         * @see com.ibm.uima.analysis_engine.annotator.JTextAnnotator#process(com.ibm.uima.jcas.impl.JCas,
061         *      com.ibm.uima.analysis_engine.ResultSpecification)
062         */
063        public void process(JCas jcas, ResultSpecification arg1) throws AnnotatorProcessException {
064            String docText = jcas.getDocumentText();
065    
066            Iterator docInfoIter = jcas.getJFSIndexRepository().getAnnotationIndex(CCPDocumentInformation.type).iterator();
067            String docID = "-1";
068            int documentCollectionID = -1;
069            if (docInfoIter.hasNext()) {
070                CCPDocumentInformation srcDocInfo = (CCPDocumentInformation) docInfoIter.next();
071                docID = srcDocInfo.getDocumentID();
072                documentCollectionID = srcDocInfo.getDocumentCollectionID();
073            }
074    
075            /* run the EI system */
076            if (DEBUG) {
077                System.out.println("Using KeX on document: [" + docID + "] " + docText);
078            }
079            List<TextAnnotation> proteinAnnotations = eiSystem.getEntitiesFromString(docText, docID);
080    
081            AnnotationSet aset = new AnnotationSet(new Integer(20),"Pre-tagged proteins and cell types","This annotation set encompasses annotations that were pre-tagged using automated methods.");
082            
083            /* Add -1 entrez_gene_id for each protein annotation */
084            for (TextAnnotation ta : proteinAnnotations) {
085                ta.addAnnotationSet(aset);
086                ta.setDocumentCollectionID(documentCollectionID);
087                ta.setDocumentID(docID);
088                ClassMention cm = ta.getClassMention();
089    //            /* make sure it is a protein */
090    //            if (cm.getMentionName().equals(ClassMentionTypes.PROTEIN)) {
091    //                SlotMention sm = cm.getSlotMentionByName(SlotMentionTypes.PROTEIN_ENTREZGENEID);
092    //                if (sm != null) {
093    //                    sm.setFirstSlotValue("-1");
094    //                } else {
095    //                    sm = new SlotMention();
096    //                    sm.setMentionName(SlotMentionTypes.PROTEIN_ENTREZGENEID);
097    //                    sm.setFirstSlotValue("-1");
098    //                    cm.addSlotMention(sm);
099    //                }
100    //            }
101            }
102    
103            
104            /* add protein annotations to the CAS */
105            UIMA_Util uimaUtil = new UIMA_Util();
106            uimaUtil.putTextAnnotationsIntoJCas(jcas, proteinAnnotations);
107    
108        }
109    }
```
